# Godey’s Lady’s Book

**Published:** 1863-10

**Authors:** 


*Godey's Lady's Book.*—By favor of the publishers of this interesting and popular magazine, we have been favored with the monthly issues, until quite recently, when for some reason the late numbers have failed to connect. The monthly numbers we have no doubt continue to please, interest and instruct those who do receive them. We do not often notice, as they deserve, these popular journals, which are sent us as a courtesy, since we hope our readers are already better acquainted with their merits than ourselves. Godey's Lady's Book is, of course, upon every lady's reading table in its advance copies.

